# Optimization of Hypothermic Protocols for Neurocognitive Preservation in Aortic Arch Surgery: A Literature Review

**DOI:** 10.3390/jcdd11080238

**Published:** 2024-08-01

**Authors:** Jordan Llerena-Velastegui, Sebastian Velastegui-Zurita, Kristina Zumbana-Podaneva, Melany Mejia-Mora, Ana Clara Fonseca Souza de Jesus, Pedro Moraes Coelho

**Affiliations:** 1 Medical School, Pontifical Catholic University of Ecuador, Quito 170525, Ecuador; sebasvelas123@hotmail.com (S.V.-Z.); kriszpmed@gmail.com (K.Z.-P.); r.bmelibrat@hotmail.com (M.M.-M.); 2 Research Center, Center for Health Research in Latin America (CISeAL), Quito 170530, Ecuador; 3 Medical School, Faculdade de Minas—FAMINAS-BH, Belo Horizonte 31744-007, Brazil; anaclarafsjj@gmail.com (A.C.F.S.d.J.); pedromoraesc1@gmail.com (P.M.C.)

**Keywords:** temperature, cerebral perfusion, cardiopulmonary bypass, aortic arch, survival rate

## Abstract

Shifts from deep to moderate hypothermic circulatory arrest (HCA) in aortic arch surgery necessitate an examination of their differential impacts on neurocognitive functions, especially structured verbal memory, given its significance for patient recovery and quality of life. This study evaluates and synthesizes evidence on the effects of deep (≤20.0 °C), low-moderate (20.1–24.0 °C), and high-moderate (24.1–28.0 °C) hypothermic temperatures on structured verbal memory preservation and overall cognitive health in patients undergoing aortic arch surgery. We evaluated the latest literature from major medical databases such as PubMed and Scopus, focusing on research from 2020 to 2024, to gather comprehensive insights into the current landscape of temperature management during HCA. This comparative analysis highlights the viability of moderate hypothermia (20.1–28.0 °C), supported by recent trials and observational studies, as a method to achieve comparable neuroprotection with fewer complications than traditional deep hypothermia. Notably, low-moderate and high-moderate temperatures have been shown to support substantial survival rates, with impacts on structured verbal memory preservation that necessitate careful selection based on individual surgical risks and patient profiles. The findings advocate for a nuanced approach to selecting hypothermic protocols in aortic arch surgeries, emphasizing the importance of tailoring temperature management to optimize neurocognitive outcomes and patient recovery. This study fills a critical gap in the literature by providing evidence-based recommendations for temperature ranges during HCA, calling for ongoing updates to clinical guidelines and further research to refine these recommendations. The implications of temperature on survival rates, complications, and success rates underpin the necessity for evolving cardiopulmonary bypass techniques and cerebral perfusion strategies to enhance patient outcomes in complex cardiovascular procedures.

## 1. Introduction

The management of temperature during hypothermic circulatory arrest (HCA) is a cornerstone of aortic arch surgery, playing a decisive role in patient outcomes, particularly in neurocognitive preservation [1]. This practice, essential for protecting cerebral function during periods of arrested blood flow, has undergone significant evolution over the past decades [2]. Traditional deep hypothermia, once the standard, is being increasingly substituted with moderate hypothermic protocols, driven by a growing body of evidence suggesting comparable efficacy in neuroprotection with potentially fewer complications and enhanced recovery profiles [3]. This review seeks to delve deeply into the nuances of how different hypothermic strategies influence structured verbal memory, a critical cognitive domain profoundly affecting patient recovery and quality of life postsurgery [4].

Structured verbal memory, essential for daily communication, personal recall, and professional functionality, emerges as a sensitive indicator of neurocognitive health following surgical interventions. It is especially susceptible to variations in the management of intraoperative temperatures [5]. Historically, deep hypothermia (≤20.0 °C) has been advocated for its robust neuroprotective properties, particularly noted for its capacity to preserve verbal memory more effectively than its moderate counterparts [6,7]. However, recent clinical trials and observational studies indicate that moderate hypothermia (20.1 °C to 28.0 °C), especially when combined with techniques such as antegrade cerebral perfusion (ACP), offers a viable alternative, challenging the supremacy of deep hypothermia by providing sufficient neuroprotection with a potentially lower risk profile [7].

The evolution from deep to moderate hypothermic practices reflects a significant shift in the paradigm of patient management during aortic arch surgery [8]. This transition is not merely a change in technique but represents a response to a critical reassessment of risk versus benefit, where the ultimate goal is to maximize patient safety and postoperative recovery [9,10]. As such, this literature review aims to systematically analyze the impacts of these hypothermic temperatures on structured verbal memory and broader cognitive functions, offering a comprehensive overview that bridges historical practices with contemporary insights.

The significance of this study lies in its potential to influence clinical practices and guidelines significantly. By elucidating which hypothermic strategies most effectively safeguard neurocognitive functions, particularly structured verbal memory, this review addresses a vital gap in the existing literature. It proposes not only to reinforce the evidence supporting moderate hypothermia’s efficacy but also to highlight areas where deep hypothermia might still hold advantages. Thus, it contributes to a more nuanced understanding of temperature management in HCA, aligning surgical practices with the latest research to optimize patient outcomes.

Furthermore, this exploration into hypothermic strategies is timely and critical. As surgical techniques and patient management protocols evolve, so too must our understanding of their long-term implications on patient health and quality of life. This study provides the groundwork for future research and clinical trials, aiming to refine and potentially redefine approaches to HCA, ensuring that every patient receives care tailored to maximize their recovery and long-term cognitive health. By advancing this field of knowledge, we contribute to the broader goals of cardiovascular surgery and patient care, ensuring that our methodologies are as safe, effective, and forward-thinking as possible.

## 2. Methodology

The purpose of this literature review is to synthesize existing research on the neurocognitive impacts of different hypothermic strategies during circulatory arrest in aortic arch surgery. Recognizing the complexity and significance of this topic, our review aims to provide a comprehensive overview that bridges historical practices with contemporary insights, particularly focusing on deep (≤20.0 °C), low-moderate (20.1–24.0 °C), and high-moderate (24.1–28.0 °C) hypothermic temperatures.

We conducted extensive searches in major medical databases, including PubMed, Scopus, and Web of Science, to identify relevant studies published between 2020 and 2024. Our search terms included “hypothermic circulatory arrest”, “aortic arch surgery”, “neurocognitive outcomes”, “structured verbal memory”, “rewarming rate”, and “temperature management”.

Studies were included if they focused on the neurocognitive outcomes of different hypothermic strategies during circulatory arrest in aortic arch surgery, investigated the impact of rewarming rates on patient outcomes, were published in peer-reviewed journals within the specified timeframe, and provided quantitative and qualitative data on survival rates, complications, and cognitive functions.

Articles were excluded if they were not published in English, did not directly relate to hypothermic circulatory arrest in aortic arch surgery, focused on pediatric populations or nonhuman subjects, or were editorial pieces without primary research data.

The selected articles were then analyzed to extract key findings related to cognitive outcomes, neuroimaging and physiological outcomes, complications, quality of life, and the rewarming process. This approach ensures a thorough and balanced representation of the current state of research in this area, providing valuable insights for clinicians and researchers alike.

## 3. Epidemiology and Demographics

The global incidence of aortic arch surgeries involving HCA varies significantly, reflecting disparities in healthcare access and surgical practices between developed and developing countries [11]. Developed nations typically report higher surgical rates and better outcomes, attributed to advanced healthcare infrastructure and the availability of specialized techniques such as ACP [12]. Conversely, in developing regions, limited access to such advanced methods may contribute to lower surgical frequencies and varied outcomes [13].

Demographic variability also plays a crucial role in the epidemiology of conditions necessitating aortic arch surgery [14]. Age, sex, and ethnicity influence the prevalence of these conditions, which subsequently affects the application of HCA [15]. For instance, younger patients and those without extensive comorbidities may experience different surgical approaches and outcomes compared to older populations or those with significant pre-existing health issues [16].

Moreover, the techniques of HCA, particularly the preferred temperature ranges for circulatory arrest, exhibit notable geographical differences [17]. While moderate hypothermia, ranging from 20.1 °C to 28.0 °C, is increasingly adopted globally, the choice between low-moderate (20.1 °C to 24.0 °C) and high-moderate (24.1 °C to 28.0 °C) temperatures often depends on the complexity of the surgical intervention and the expected duration of the arrest [7]. Technological availability, adherence to clinical guidelines, and surgeon preferences are key factors influencing these variations [18].

Patient-specific risk factors such as pre-existing neurological conditions and overall cardiovascular health are significant determinants of the choice of hypothermic regime [19]. These factors not only influence the surgical approach but also affect postoperative outcomes, particularly neurocognitive functions [7,19]. For example, patients with a history of neurological issues may be more vulnerable to adverse outcomes associated with deeper levels of hypothermia [16].

Temporal trends in the use of HCA techniques have evolved, with a noticeable shift from deep hypothermia (≤20.0 °C) to moderate hypothermia (20.1 °C to 28.0 °C) over recent years [7]. This shift is supported by clinical trials and observational studies which suggest comparable neurocognitive and neuroimaging outcomes among patients treated under different hypothermic conditions [7,20]. The evolution in practice is also influenced by landmark studies which underscore the noninferiority of low-moderate hypothermia in preserving structured verbal memory compared to deeper hypothermic conditions, alongside similar mortality and morbidity rates across varying degrees of hypothermia [21].

These insights into the epidemiology and demographics of aortic arch surgery using hypothermic circulatory arrest provide a comprehensive understanding of the factors that guide surgical decisions and influence patient outcomes, highlighting the need for continual evaluation and adaptation of surgical techniques to optimize care across diverse patient populations.

## 4. Temperature Regimes and Their Effects

In the realm of aortic arch surgery, the strategic use of HCA plays a pivotal role in optimizing patient outcomes by mitigating neurological risks during periods of suspended blood flow [22]. This narrative delineates the specific hypothermic temperature regimes employed (deep, low-moderate, and high-moderate), highlighting the evolution of these techniques, their clinical justifications, and the associated neurocognitive outcomes [23].

The historical advent of HCA techniques has been marked by the use of various cooling methods such as ice slush, cooling blankets, and advanced cardiopulmonary bypass configurations to achieve desired hypothermic depths [24]. These methods have evolved significantly, facilitated by technological advancements in monitoring systems that allow for precise control of body temperature [24,25]. The clinical rationale for employing specific temperature ranges largely stems from the need to balance neuroprotection with the risks inherent in hypothermic treatment [26]. Studies consistently explore these temperature-dependent strategies to evaluate their efficacy in preserving cognitive functions, particularly during the vulnerable period of circulatory arrest [7].

Traditionally, deep hypothermia has been implemented to reduce metabolic rates to a bare minimum, thereby extending the safe period for surgical intervention without blood flow [27]. By maintaining temperatures at or below 20.0 °C, deep hypothermia offers substantial neuroprotection, believed to be due to significant reductions in cerebral metabolic demands [7]. This approach, however, carries potential complications such as coagulopathy, increased infection rates, and prolonged recovery times [25]. Despite these risks, it has been particularly noted for its effectiveness in preserving structured verbal memory and other critical neurological functions, which underscores its continued use in cases where maximal brain protection is imperative [28].

Shifting towards less intensive cooling strategies, low-moderate hypothermia, which involves maintaining body temperatures between 20.1 °C and 24.0 °C, represents a methodological adjustment aimed at reducing the complications associated with deeper hypothermic states [7]. This temperature range is chosen to provide adequate neuroprotection while minimizing adverse effects such as shivering and arrhythmias, which are more prevalent at lower temperatures [7,29]. Clinical studies evaluating this regime suggest that, while it generally maintains noninferiority to deep hypothermia in terms of overall cognitive changes, it may not preserve structured verbal memory as effectively [30].

The high-moderate hypothermia range, maintaining temperatures between 24.1 °C and 28.0 °C, has emerged as a preferable option in some surgical practices due to its even lower risk profile [7]. The clinical investigations into this temperature spectrum reveal that it can sustain neurocognitive outcomes comparable to those of deeper hypothermia, with added benefits including a reduced incidence of complications typically associated with colder temperatures [3]. This regime is often selected based on the complexity of the surgical intervention and the anticipated duration of hypothermia, reflecting a tailored approach to patient care in aortic arch surgery [31].

This evolution from deep to moderate hypothermia in HCA reflects a paradigm shift towards optimizing patient safety and outcome efficacy [8]. With advancements in surgical techniques and temperature management technology, the field continues to refine its approach to balancing the neuroprotective benefits of hypothermia against its potential risks [7,8]. The selection of hypothermic depth is thus increasingly guided by a comprehensive assessment of patient-specific factors and surgical complexities, ensuring that the chosen regime maximizes benefits while minimizing risks.

## 5. Cognitive Outcomes

In aortic arch surgery, the application of HCA at varying temperatures—deep (≤20.0 °C), low-moderate (20.1–24.0 °C), and high-moderate (24.1–28.0 °C)—has profound implications for cognitive outcomes [7]. This examination synthesizes the impacts of these hypothermic conditions on global cognitive function and structured verbal memory, assessing the efficacy of temperature-specific protocols in safeguarding cognitive integrity postsurgery [7,32].

Emerging research indicates a nuanced relationship between hypothermic depth and cognitive recovery postsurgery [7,16]. Observational data and clinical trials have increasingly supported a shift from deep to moderate hypothermia, particularly when used in conjunction with ACP [7,33]. For instance, a recent multicenter trial assessed the neurocognitive and neuroimaging outcomes of patients under these varied hypothermic conditions. The results revealed that low-moderate and high-moderate hypothermia demonstrated noninferiority to deep hypothermia concerning global cognitive change scores from baseline to four weeks postoperatively [7]. This suggests that moderate hypothermia, spanning from 20.1 to 28.0 °C, is a viable alternative to deep hypothermia, potentially reducing associated risks without compromising cognitive outcomes [7,12].

Commonly utilized assessments such as the mini-mental state examination (MMSE) and the Montreal cognitive assessment (MoCA) have provided quantitative measures of cognitive function across these studies [34]. However, confounding factors, like the duration of circulatory arrest and variations in patient age, necessitate careful interpretation of data, as these elements significantly influence recovery trajectories [25,34].

Structured verbal memory, a critical component of postoperative cognitive recovery, appears particularly sensitive to variations in hypothermic depth. Deep hypothermia (≤20.0 °C) has been shown to preserve structured verbal memory more effectively than its moderate counterparts [7]. This finding is crucial, as structured verbal memory impacts a patient’s ability to organize and recall information, influencing quality of life and postoperative adjustment [26]. Standardized tests such as the California verbal learning test and the verbal paired associates subtest from the Wechsler memory scale have been instrumental in documenting these differences [35,36]. The specificity of deep hypothermia in safeguarding this cognitive domain highlights its continued relevance, especially in complex surgical scenarios where prolonged circulatory arrest is anticipated (Table 1) [7].

Beyond global cognitive function and structured verbal memory, other cognitive domains such as executive function, attention, and perceptual speed also warrant consideration [37]. Various cognitive tests, including the Trail Making test for executive function and attention and the digit symbol substitution test for perceptual speed, have been employed to evaluate these aspects under different hypothermic conditions [32,38]. The findings across studies suggest that, while moderate hypothermia provides adequate protection for general cognitive functions, the precision in maintaining specific cognitive abilities can vary with temperature depths [39]. These outcomes are critical for informing postoperative rehabilitation strategies and enhancing patient recovery profiles [40].

In conclusion, the strategic application of HCA at tailored temperature ranges offers significant neuroprotective benefits, with implications for a broad spectrum of cognitive outcomes. While deep hypothermia remains superior for the preservation of structured verbal memory, the advancements in moderate hypothermia protocols present a safer, equally efficacious alternative for protecting overall cognitive function. As such, clinical decisions regarding the optimal hypothermic depth should consider both the specific cognitive risks associated with the surgical procedure and the broader implications for patient recovery and quality of life.

## 6. Neuroimaging and Physiological Outcomes

In aortic arch surgery, HCA plays a pivotal role in mitigating the risk of neurologic damage by reducing metabolic demands during periods of circulatory standstill [2]. This critical review explores the neuroimaging and physiological outcomes of various HCA temperature protocols, particularly focusing on magnetic resonance imaging (MRI) and computed tomography (CT) findings, along with assessments of functional brain connectivity [16].

MRI and CT scans are instrumental in detecting and analyzing the structural brain changes postoperatively across varying hypothermic conditions [41]. Deep hypothermia, defined as temperatures ≤20.0 °C, has historically been utilized to provide maximal neuroprotection, which is evident in the reduced incidence of ischemic lesions and hemorrhagic events as visualized through these imaging techniques. In comparison, low-moderate hypothermia (20.1–24.0 °C) and high-moderate hypothermia (24.1–28.0 °C) have shown similar structural outcomes on neuroimaging but with less severe alterations, suggesting a potential reduction in hypothermia-associated complications such as coagulopathy and infection rates, which can influence neurologic outcomes [15,41].

The relationship between the depth of hypothermia and structural brain damage observed in neuroimaging reveals that, while deeper hypothermia may protect against gross structural injuries, it does not entirely eliminate subtle changes such as minor ischemic strokes or diffuse axonal injury [42]. This indicates a complex interaction between temperature depth and neuroprotection, where moderate temperatures might offer a balanced approach by providing adequate neuroprotection with fewer complications [43].

Turning to functional brain connectivity, advanced neuroimaging techniques such as functional MRI (fMRI) have provided insights into how different hypothermic protocols affect neural networks involved in cognitive functions [13,41]. Studies have documented that temperature-induced alterations in brain connectivity can significantly impact cognitive recovery postsurgery [44]. For instance, changes in the default mode network, sensorimotor network, and executive control network during HCA have been correlated with long-term cognitive outcomes [45]. Data suggest that, while deep hypothermia might preserve certain aspects of network connectivity related to memory and executive functions, moderate hypothermia sufficiently maintains overall connectivity, thus supporting adequate cognitive recovery [44,45].

In synthesis, the existing literature underscores the complexity of choosing an optimal hypothermic depth. While deep hypothermia provides extensive neuroprotection, it comes with increased risks of adverse effects. Moderate hypothermia, ranging from 20.1 °C to 28.0 °C, appears to offer a pragmatic balance by ensuring substantial neuroprotection with fewer complications, aligning with recent shifts in clinical practice towards milder cooling protocols. The implications of these findings are critical for refining surgical practices and optimizing patient outcomes in aortic arch surgery involving HCA.

## 7. Complications and Quality of Life

In aortic arch surgery, employing HCA at varying temperature levels crucially impacts postoperative complications and long-term quality of life [46]. This critical review synthesizes current research findings to elucidate the relationship between different hypothermic temperatures and their respective outcomes, focusing on deep, low-moderate, and high-moderate hypothermic conditions [7].

Postoperative complications vary significantly with the depth of hypothermia [26]. Deep hypothermia (≤20.0 °C) is particularly associated with a higher incidence of coagulopathies, infections, and prolonged patient sedation [7,47]. Cardiac complications, such as arrhythmias and myocardial stunning, are more pronounced due to the increased physiological stress on the heart at these lower temperatures [48]. Neurologically, patients are at a greater risk for delirium, stroke, or transient ischemic attacks, owing to the extended duration of circulatory arrest required [16].

In contrast, low-moderate hypothermia (20.1–24.0 °C) strikes a balance between reducing the severity of deep hypothermia-related complications and maintaining adequate neuroprotection [7,16]. This temperature range tends to have milder instances of coagulopathies and infections and shows a decreased frequency and severity of cardiac complications compared to deep hypothermia [47].

High-moderate hypothermia (24.1–28.0 °C) potentially leads to the fewest hypothermia-related complications [7]. This warmer range is associated with reduced instances of shivering, lower risks of coagulopathy, and diminished cardiac stress [47]. Neurological complications, including cognitive deficits and sensorimotor impairments, appear to vary, showing potential differences from those observed at lower temperature conditions [16,47].

The long-term quality of life implications based on these different hypothermic protocols are profound. Each temperature range uniquely affects cognitive outcomes, with deep hypothermia better preserving structured verbal memory, which is crucial for daily activities and social interactions. However, low-moderate and high-moderate hypothermia might allow for quicker cognitive recovery and less severe long-term neurological deficits [7].

Physical health and mobility postsurgery are directly influenced by the chosen hypothermic strategy. Patients experiencing fewer complications from higher temperature protocols generally exhibit better physical recovery, less chronic pain, and reduced physical disabilities. This directly translates into improved daily functioning and quality of life [33].

Emotional and psychological health are also impacted by the recovery trajectory. The prevalence of postoperative depression or anxiety can be influenced by prolonged recovery periods and significant shifts in cognitive and physical capabilities. Patients maintained at warmer hypothermic levels often face shorter and potentially less traumatic recovery periods, which could mitigate some emotional and psychological challenges [49].

Additionally, social reintegration and the need for support services vary with the severity of complications and cognitive impairments. Those undergoing less severe hypothermic protocols may require less extensive rehabilitation services and community support, facilitating a smoother transition back to normal life [50].

In conclusion, the selection of hypothermic temperature during HCA is a critical determinant of both immediate postoperative outcomes and long-term quality of life. The findings suggest that, while deep hypothermia provides substantial neuroprotection, it is associated with higher risks and complications. Low-moderate and high-moderate hypothermia offer a more balanced approach, potentially leading to better overall outcomes and quality of life, underscoring the need for tailored hypothermic strategies based on individual patient profiles and surgical specifics.

## 8. Gaps in the Literature

The exploration of HCA in aortic arch surgery and its impacts on structured verbal memory and other cognitive functions has garnered significant attention. However, several gaps in the existing literature curtail the comprehensive understanding and application of findings to clinical practice. This section delineates these lacunae, emphasizing the need for methodological refinement and extended scope of research to fortify our understanding of HCA’s cognitive outcomes [51].

A notable deficiency in current research is the generalizability of existing studies, constrained by a lack of diversity in patient populations. Many studies predominantly include homogeneous groups, often limited to specific demographic profiles [44]. This raises concerns about the applicability of findings across diverse global populations, which may exhibit different risk profiles and outcomes. There is a pressing need for research that encompasses a broader spectrum of ethnicities, ages, and comorbid conditions, which would enhance the external validity of the findings and facilitate their application in varied clinical settings worldwide [52].

Additionally, the variability in surgical techniques and perioperative care across different institutions and regions introduces another layer of complexity. This variability is infrequently accounted for in the research, which may confound the results and limit their applicability to broader surgical practices. Standardization of surgical techniques and perioperative measures is crucial for reducing variability and enhancing the reliability of study outcomes across different settings [53].

Methodologically, several studies suffer from inherent weaknesses that undermine their scientific rigor. Small sample sizes are a prevalent issue, restricting the statistical power and generalizability of the results. This limitation necessitates the advocacy for larger, multicenter trials that can yield more robust data and provide definitive conclusions about the effects of hypothermic temperatures on cognitive outcomes [54]. Furthermore, the frequent absence of randomization or control groups in many studies on hypothermia limits the ability to draw causal inferences, potentially introducing bias. The implementation of randomized controlled trials would substantially mitigate these limitations, providing a clearer delineation of the effects of temperature management [47,54].

Another critical methodological limitation in hypothermia research is the predominance of short follow-up periods, which are insufficient to assess the long-term effects on neurocognitive functions. Extending these follow-ups to several months or years would enable a thorough evaluation of lasting impacts, potentially delayed complications, and cognitive recoveries [55]. Moreover, inconsistencies in cognitive assessments and outcome measures across studies hinder reliable comparisons and systematic reviews. Adopting standardized testing protocols would facilitate more consistent meta-analyses and enhance the clinical applicability of the evidence. A notable deficiency is the limited focus on long-term cognitive outcomes; most studies concentrate on immediate and short-term effects postsurgery, neglecting the prolonged impacts of various hypothermic protocols on higher-order cognitive functions such as memory, problem-solving, and executive abilities. The scarcity of longitudinal studies that track cognitive recovery over extended periods postsurgery represents a significant gap, leading to an incomplete understanding of the enduring impacts of hypothermia on cognitive health [38].

Addressing these gaps and methodological issues in future research is imperative for generating comprehensive, reliable, and applicable data that can significantly influence clinical practices and optimize patient outcomes in the realm of hypothermic circulatory arrest during aortic arch surgery. Another critical aspect to consider is the rewarming rate after circulatory arrest. The literature provides varying recommendations on the optimal temperature range to maintain and the duration for rewarming to normothermia. Studies such as those by Rungatscher et al. [56] and Linardi et al. [57] highlight the importance of careful temperature management during the rewarming phase. Rungatscher et al. found that postoperative hyperthermia (>37 °C) is associated with higher risks of 30-day mortality, stroke incidence, and poor neurological outcomes compared to normothermia. Linardi et al. demonstrated that slow rewarming significantly reduces neuroinflammation, enhances cerebral perfusion, and reduces cerebral edema, suggesting that slow rewarming might be superior to fast rewarming in improving neurological outcomes. Therefore, future research should also focus on establishing clear guidelines for rewarming protocols to enhance patient safety and recovery.

## 9. Future Research Directions

To advance the field of HCA in aortic arch surgery, several research initiatives are proposed, targeting both methodological enhancements and a broader understanding of clinical impacts. These recommendations aim to refine current practices and improve patient outcomes through precision in temperature management and technique application [58].

Prospective multicenter trials are imperative to conduct large-scale, prospective multicenter trials that capture diverse patient demographics and healthcare settings. These studies should prioritize evaluating long-term cognitive outcomes, with an emphasis on structured verbal memory and executive functions across various hypothermic temperatures. By standardizing protocols and methodologies, such trials can provide definitive insights into the optimal temperature ranges for neuroprotection and potentially reshape clinical approaches to HCA [59].

Mechanistic studies delve deeper into the biochemical and physiological mechanisms that underlie temperature-related neuroprotection. Investigating the cellular and molecular responses of brain tissue to varying degrees of cooling can elucidate pathways that either confer neuroprotection or contribute to neurodegeneration. Utilizing advanced imaging techniques, biomarker analyses, and genetic profiling will enhance our understanding and guide targeted therapeutic strategies [60].

Comparative effectiveness research evaluates the practical outcomes of different hypothermic protocols in routine clinical practice, assessing both clinical and economic factors. Such studies are crucial for determining cost-effective strategies that do not compromise the quality of care, thereby informing policy and practice to optimize resource utilization in healthcare settings [26].

Quality-of-life studies undertake comprehensive investigations into the quality of life and functional status of patients at various intervals postsurgery. These studies will not only illuminate the recovery trajectory but also help identify factors influencing long-term well-being and social reintegration after surgery. Understanding these dynamics is essential for patient counseling and developing support systems tailored to patient needs [49].

Implications for policy and practice include future research findings that should be integrated into existing clinical guidelines for aortic arch surgery. This integration must consider new evidence on optimal hypothermia levels, patient selection criteria, and refined surgical techniques to enhance patient safety and outcomes [16].

There is a need for comprehensive decision-making frameworks that incorporate individual patient factors such as age, health conditions, and personal risk profiles. These frameworks will enable personalized treatment plans, improving outcomes and minimizing complications [61].

Training and education ensure that healthcare providers are well-versed in the latest research and technological advancements in HCA. Continuous education programs and training workshops will help clinicians apply the most recent findings effectively and adaptively in clinical settings [11].

Patient education and informed consent that enhance patient education about the risks and benefits of various hypothermic protocols is crucial. Improved educational efforts will support a more informed consent process, align patient expectations with potential outcomes, and foster patient engagement in their care processes [49].

Through these directed research efforts and their integration into clinical practice, the application of hypothermic circulatory arrest in aortic arch surgery can be optimized, leading to enhanced patient outcomes and more effective use of healthcare resources. These initiatives will collectively contribute to a more evidence-based approach to managing patients undergoing complex surgical interventions involving HCA.

## 10. Conclusions

This study significantly advances the field of cardiovascular surgery by elucidating the differential impacts of deep, low-moderate, and high-moderate hypothermic temperatures on structured verbal memory and overall neurocognitive outcomes in aortic arch surgery. It establishes moderate hypothermia as a viable and often preferable alternative to deep hypothermia, offering substantial neuroprotection with fewer complications and quicker recovery, which challenges existing paradigms and encourages the re-evaluation of clinical protocols. This contribution is pivotal, as it not only enhances patient safety and postoperative recovery but also informs tailored therapeutic strategies that consider individual patient profiles and surgical complexities. By fostering a nuanced understanding of hypothermic circulatory arrest, this research underpins future innovations in surgical practice and patient care, advocating for an evidence-based approach to optimizing neurocognitive outcomes in complex cardiovascular procedures.

## Figures and Tables

**Table 1:** Summary of principal findings and temperature ranges in cited studies.

Study	Temperature Range	Percentage of Neurological Impairment	Main Complications
Wang et al. (2020) [1]	≤20.0 °C	15%	Coagulopathy, infections
Ghia et al. (2023) [2]	20.1 °C to 24.0 °C	10%	Mild coagulopathies, infections
Shimamura et al. (2023) [3]	24.1 °C to 28.0 °C	8%	Reduced shivering, lower coagulopathy risks

## Data Availability

All data generated or analyzed during this study are included in this published article. Further inquiries should be directed to the corresponding author.

## References

[1] Wang X., Yang F., Zhu J., Liu Y., Sun L., Hou X. (2020). Aortic arch surgery with hypothermic circulatory arrest and unilateral antegrade cerebral perfusion: Perioperative outcomes. J. Thorac. Cardiovasc. Surg..

[2] Ghia S., Savadjian A., Shin D., Diluozzo G., Weiner M.M., Bhatt H.V. (2023). Hypothermic Circulatory Arrest in Adult Aortic Arch Surgery: A Review of Hypothermic Circulatory Arrest and its Anesthetic Implications. J. Cardiothorac. Vasc. Anesth..

[3] Shimamura J., Yokoyama Y., Kuno T., Fujisaki T., Fukuhara S., Takayama H., Ota T., Chu M.W. (2023). Systematic review and network meta-analysis of various nadir temperature strategies for hypothermic circulatory arrest for aortic arch surgery. Asian Cardiovasc. Thorac. Ann..

[4] Stewart J.A., Särkelä M.O.K., Salmi T., Wennervirta J., Vakkuri A.P., Vainikka T.L.S., Suojaranta R., Mäki K., Ilkka V.H., Viertiö-Oja H. (2020). Noninvasive Neuromonitoring of Hypothermic Circulatory Arrest in Aortic Surgery. Scand. J. Surg..

[5] Sato H., Kawaharada N., Fukada J., Nakanishi K., Mikami T., Shibata T., Harada R., Naraoka S., Kamada T., Tamiya Y. (2022). Estimation Model for Hypothermic Circulatory Arrest Time to Predict Risk in Total Arch Replacement. Ann. Thorac. Surg..

[6] Seese L., Chen E.P., Badhwar V., Thibault D., Habib R.H., Jacobs J.P., Thourani V., Bakaeen F., O’Brien S., Jawitz O.K. (2023). Optimal circulatory arrest temperature for aortic hemiarch replacement with antegrade brain perfusion. J. Thorac. Cardiovasc. Surg..

[7] Hughes G.C., Chen E.P., Browndyke J.N., Szeto W.Y., DiMaio J.M., Brinkman W.T., Gaca J.G., Blumenthal J.A., Karhausen J.A., Bisanar T. (2024). Cognitive Effects of Body Temperature during Hypothermic Circulatory Arrest Trial (GOT ICE): A Randomized Clinical Trial Comparing Outcomes after Aortic Arch Surgery. Circulation.

[8] Urbanski P.P., Lenos A., Bougioukakis P., Neophytou I., Zacher M., Diegeler A. (2012). Mild-to-moderate hypothermia in aortic arch surgery using circulatory arrest: A change of paradigm?. Eur. J. Cardio-Thoracic Surg..

[9] Li H., Yu H., Dong N., Wu L. (2021). A Modified Hypothermic Circulatory Arrest Technique Improves Early and Near-Midterm Results in Patients with Acute Type A Aortic Dissection. Heart Surg. Forum.

[10] Jiang H., Liu Y., Yang Z., Ge Y., Du Y. (2021). Mild Hypothermic Circulatory Arrest with Lower Body Perfusion for Total Arch Replacement via Upper Hemisternotomy in Acute Type A Dissection. Heart Surg. Forum.

[11] Mazine A., Stevens L.M., Ghoneim A., Chung J., Ouzounian M., Dagenais F., El-Hamamsy I., Boodhwani M., Bozinovski J., Peterson M.D. (2019). Developing skills for thoracic aortic surgery with hypothermic circulatory arrest. J. Thorac. Cardiovasc. Surg..

[12] Wang L., Zhong G., Lv X., Dong Y., Hou Y., Chen L. (2024). Clinical outcomes of mild versus moderate hypothermic circulatory arrest with antegrade cerebral perfusion in adult aortic arch surgery: A systematic review and meta-analysis. Perfusion.

[13] Gutsche J.T., Ghadimi K., Patel P.A., Robinson A.R., Lane B.J., Szeto W.Y., Augoustides J.G. (2014). New frontiers in aortic therapy: Focus on deep hypothermic circulatory arrest. J. Cardiothorac. Vasc. Anesth..

[14] Sethi S., Parekh U. (2024). Aortic Arch Aneurysm 2023 Jul 24. StatPearls [Internet].

[15] Dong S.B., Zhang K., Zhu K., Wang L.-F., Zheng J., Li J.-R., Liu Y.-M., Sun L.-Z., Pan X.-D. (2021). Mild hypothermic circulatory arrest with selective cerebral perfusion in open arch surgery. J. Thorac. Dis..

[16] Salem M., Friedrich C., Thiem A., Salem M.A., Puehler T., Rusch R., Berndt R., Cremer J., Haneya A. (2020). Effect of moderate hypothermic circulatory arrest on neurological outcomes in elderly patients undergoing replacement of the thoracic aorta. Egypt. Heart J..

[17] Sato H., Iba Y., Kawaharada N., Fukada J., Iwashiro Y., Tsushima S., Hosaka I., Okawa A., Shibata T., Nakazawa J. (2023). Temperature analysis of aortic repair with hypothermic circulatory arrest to quantify the injury by cooling. Interdiscip. Cardiovasc. Thorac. Surg..

[18] Arnaoutakis G.J., Ogami T., Bobba C.M., Serna-Gallegos D., Brown J.A., Jeng E.I., Martin T.D., Beaver T.M., Yousef S., Navid F. (2022). Cerebral protection using deep hypothermic circulatory arrest versus retrograde cerebral perfusion for aortic hemiarch reconstruction. J. Card. Surg..

[19] Hirsch K.G., Abella B.S., Amorim E., Bader M.K., Barletta J.F., Berg K., Callaway C.W., Friberg H., Gilmore E.J., Greer D.M. (2024). Critical Care Management of Patients After Cardiac Arrest: A Scientific Statement from the American Heart Association and Neurocritical Care Society. Circulation.

[20] Tanaka A., Chehadi M., Smith H.N., Hassan M., Sandhu H.K., Miller C.C., Safi H.J., Estrera A.L. (2023). Deep Hypothermic Circulatory Arrest with Retrograde Cerebral Perfusion: How Long Is Safe?. Ann. Thorac. Surg..

[21] Bancone C., Della Corte A., Lo Presti F., Ashurov R., Sica G., Palmieri L., Di Fraia R., De Feo M. (2024). Open aortic arch repair without circulatory arrest by frozen elephant trunk in Ishimaru zone 0. J. Cardiothorac. Surg..

[22] Samanidis G., Kanakis M., Kolovou K., Perreas K. (2022). Does deep hypothermic circulatory arrest with versus without retrograde cerebral perfusion affect the outcomes after proximal aortic arch aneurysm and acute type A aortic dissection repair? Different pathologies and cerebral protection techniques with similar results. J. Card. Surg..

[23] Lou X., Chen E.P. (2021). Goal-directed cerebral perfusion in aortic arch surgery: Scientific leap or hype?. Asian Cardiovasc. Thorac. Ann..

[24] Englum B.R., He X., Gulack B.C., Ganapathi A.M., Mathew J.P., Brennan J.M., Reece T.B., Keeling W.B., Leshnower B.G., Chen E.P. (2017). Hypothermia and cerebral protection strategies in aortic arch surgery: A comparative effectiveness analysis from the STS Adult Cardiac Surgery Database. Eur. J. Cardiothorac. Surg..

[25] Tian D.H., Wan B., Bannon P.G., Misfeld M., LeMaire S.A., Kazui T., Kouchoukos N.T., Elefteriades J.A., Bavaria J., Coselli J.S. (2013). A meta-analysis of deep hypothermic circulatory arrest versus moderate hypothermic circulatory arrest with selective antegrade cerebral perfusion. Ann. Cardiothorac. Surg..

[26] Ziganshin B.A., Elefteriades J.A. (2013). Deep hypothermic circulatory arrest. Ann. Cardiothorac. Surg..

[27] Ramadoss N., Dharmapuram A.K., Goutami V., Verma S. (2021). Avoiding use of total circulatory arrest in the practice of congenital heart surgery. Indian J. Thorac. Cardiovasc. Surg..

[28] Gocoł R., Hudziak D., Bis J., Mendrala K., Morkisz Ł., Podsiadło P., Kosiński S., Piątek J., Darocha T. (2021). The Role of Deep Hypothermia in Cardiac Surgery. Int. J. Environ. Res. Public Health.

[29] Preventza O., Coselli J.S., Akvan S., Kashyap S.A., Garcia A., Simpson K.H., Price M.D., Mayor J., de la Cruz K.I., Cornwell L.D. (2017). The impact of temperature in aortic arch surgery patients receiving antegrade cerebral perfusion for >30 minutes: How relevant is it really?. J. Thorac. Cardiovasc. Surg..

[30] Chau K.H., Friedman T., Tranquilli M., Elefteriades J.A. (2013). Deep hypothermic circulatory arrest effectively preserves neurocognitive function. Ann. Thorac. Surg..

[31] Hagl C., Khaladj N., Karck M., Kallenbach K., Leyh R., Winterhalter M., Haverich A. (2003). Hypothermic circulatory arrest during ascending and aortic arch surgery: The theoretical impact of different cerebral perfusion techniques and other methods of cerebral protection. Eur. J. Cardiothorac. Surg..

[32] Hagberg G., Ihle-Hansen H., Sandset E.C., Jacobsen D., Wimmer H., Ihle-Hansen H. (2022). Long Term Cognitive Function after Cardiac Arrest: A Mini-Review. Front. Aging Neurosci..

[33] Kamenskaya O.V., Klinkova A.S., Chernyavsky A.M., Lomivorotov V.V., Meshkov I.O., Karaskov A.M. (2017). Deep Hypothermic Circulatory Arrest vs. Antegrade Cerebral Perfusion in Cerebral Protection during the Surgical Treatment of Chronic Dissection of the Ascending and Arch Aorta. J. Extra Corpor. Technol..

[34] Koller A.C., Rittenberger J.C., Repine M.J., Morgan P.W., Kristan J., Callaway C.W. (2017). Comparison of three cognitive exams in cardiac arrest survivors. Resuscitation.

[35] Svensson L.G., Nadolny E.M., Penney D.L., Jacobson J., A Kimmel W., Entrup M.H., D’agostino R.S. (2001). Prospective randomized neurocognitive and S-100 study of hypothermic circulatory arrest, retrograde brain perfusion, and antegrade brain perfusion for aortic arch operations. Ann. Thorac. Surg..

[36] Reich D.L., Uysal S., Ergin M.A., Griepp R.B. (2001). Retrograde cerebral perfusion as a method of neuroprotection during thoracic aortic surgery. Ann. Thorac. Surg..

[37] Lim C., Alexander M.P., LaFleche G., Schnyer D.M., Verfaellie M. (2004). The neurological and cognitive sequelae of cardiac arrest. Neurology.

[38] Blennow Nordström E., Vestberg S., Evald L., Mion M., Segerström M., Ullén S., Bro-Jeppesen J., Friberg H., Heimburg K., Grejs A.M. (2023). Neuropsychological outcome after cardiac arrest: Results from a sub-study of the targeted hypothermia versus targeted normothermia after out-of-hospital cardiac arrest (TTM2) trial. Crit. Care.

[39] Wang H., Wang B., Normoyle K.P., Jackson K., Spitler K., Sharrock M.F., Miller C.M., Best C., Llano D., Du R. (2014). Brain temperature and its fundamental properties: A review for clinical neuroscientists. Front. Neurosci..

[40] Dong S.B., Xiong J.X., Zhang K., Zheng J., Xu S.-D., Liu Y.-M., Sun L.-Z., Pan X.-D. (2020). Different hypothermic and cerebral perfusion strategies in extended arch replacement for acute type a aortic dissection: A retrospective comparative study. J. Cardiothorac. Surg..

[41] Sinha A., Bhatia V., Debi U., Singh L., Bhalla A., Sandhu M. (2019). Imaging in Circulatory Arrest: Lessons to be Learned. J. Clin. Imaging Sci..

[42] Zhuang J., Lin X., Lin J., Yu S., Dai S., Yan L., Chen Y., Wang R. (2022). The Evaluation Value of Diffusion-Weighted Imaging for Brain Injury in Patients after Deep Hypothermic Circulatory Arrest. Contrast Media Mol. Imaging.

[43] Manoly I., Uzzaman M., Karangelis D., Kuduvalli M., Georgakarakos E., Quarto C., Ravishankar R., Mitropoulos F., Nasir A. (2022). Neuroprotective strategies with circulatory arrest in open aortic surgery—A meta-analysis. Asian Cardiovasc. Thorac. Ann..

[44] Kim T.H., Oh J., Lee H., Kim M.S., Sim S.-A., Min S., Song S.-W., Kim J.-J. (2023). The impact of circulatory arrest with selective antegrade cerebral perfusion on brain functional connectivity and postoperative cognitive function. Sci. Rep..

[45] Ghincea C.V., Anderson D.A., Ikeno Y., Roda G.F., Eldeiry M., Bronsert M.R., Aunkst K., Fullerton D.A., Reece T.B., Aftab M. (2021). Utility of neuromonitoring in hypothermic circulatory arrest cases for early detection of stroke: Listening through the noise. J. Thorac. Cardiovasc. Surg..

[46] Stewart J.A., Ilkka V.H., Jokinen J.J., Vakkuri A.P., Suojaranta R.T., Wennervirta J., Salminen U.S. (2018). Long-Term Survival and Quality of Life after Hypothermic Circulatory Arrest in Aortic Surgery. Scand. J. Surg..

[47] Ise H., Kitahara H., Oyama K., Takahashi K., Kanda H., Fujii S., Kunisawa T., Kamiya H. (2020). Hypothermic circulatory arrest induced coagulopathy: Rotational thromboelastometry analysis. Gen. Thorac. Cardiovasc. Surg..

[48] Filseth O.M., How O.J., Kondratiev T., Gamst T.M., Tveita T. (2010). Post-hypothermic cardiac left ventricular systolic dysfunction after rewarming in an intact pig model. Crit. Care.

[49] Agarwal S., Birk J.L., Abukhadra S.L., Rojas D.A., Cornelius T.M., Bergman M., Chang B.P., Edmondson D.E., Kronish I.M. (2022). Psychological Distress After Sudden Cardiac Arrest and Its Impact on Recovery. Curr. Cardiol. Rep..

[50] Mesnard T., Dubosq M., Pruvot L., Azzaoui R., Patterson B.O., Sobocinski J. (2023). Benefits of Prehabilitation before Complex Aortic Surgery. J. Clin. Med..

[51] Zhu P., Du S., Chen S., Zheng S., Hu Y., Liu L., Zheng S. (2018). The role of deep hypothermic circulatory arrest in surgery for renal or adrenal tumor with vena cava thrombus: A single-institution experience. J. Cardiothorac. Surg..

[52] Jacobs C.S., Beers L., Park S., Scirica B., Henderson G.V., Hsu L., Bevers M., Dworetzky B.A., Lee J.W. (2020). Racial and Ethnic Disparities in Postcardiac Arrest Targeted Temperature Management Outcomes. Crit. Care Med..

[53] Balan R., Soso P., Massoudy P., Proschek T., Kurre W., Mogilansky C. (2023). A Strategy for Minimizing Circulatory Arrest Duration in Complex Aortic Arch Procedures. Medicina.

[54] Poon S.S., Estrera A., Oo A., Field M. (2016). Is moderate hypothermic circulatory arrest with selective antegrade cerebral perfusion superior to deep hypothermic circulatory arrest in elective aortic arch surgery?. Interact. Cardiovasc. Thorac. Surg..

[55] Brown J.A., Navid F., Serna-Gallegos D., Aranda-Michel E., Wang Y., Bianco V., Sultan I. (2023). Long-term outcomes of hemiarch replacement with hypothermic circulatory arrest and retrograde cerebral perfusion. J. Thorac. Cardiovasc. Surg..

[56] Rungatscher A., Luciani G.B., Linardi D., Milani E., Gottin L., Walpoth B., Faggian G. (2017). Temperature Variation After Rewarming from Deep Hypothermic Circulatory Arrest Is Associated with Survival and Neurologic Outcome. Ther. Hypothermia Temp. Manag..

[57] Linardi D., Walpoth B., Mani R., Murari A., Tessari M., Hoxha S., Anderloni M., Decimo I., Dolci S., Nicolato E. (2020). Slow versus fast rewarming after hypothermic circulatory arrest: Effects on neuroinflammation and cerebral oedema. Eur. J. Cardiothorac. Surg..

[58] Tan S.Z., Singh S., Austin N.J., Palanca J.A., Jubouri M., Girardi L.N., Chen E.P., Bashir M. (2022). Duration of deep hypothermic circulatory arrest for aortic arch surgery: Is it a myth, fiction, or scientific leap?. J. Cardiovasc. Surg..

[59] Abjigitova D., Notenboom M.L., Veen K.M., van Tussenbroek G., Bekkers J.A., Mokhles M.M., Bogers A.J.J.C. (2022). Optimal temperature management in aortic arch surgery: A systematic review and network meta-analysis. J. Card. Surg..

[60] Gupta P., Harky A., Jahangeer S., Adams B., Bashir M. (2018). Varying Evidence on Deep Hypothermic Circulatory Arrest in Thoracic Aortic Aneurysm Surgery. Tex. Heart Inst. J..

[61] Guo M.H., Stevens L.M., Chu M.W.A., Hage A., Chung J., El-Hamamsy I., Dagenais F., Peterson M., Herman C., Bozinovski J. (2024). Risk score for arch reconstruction under circulatory arrest with hypothermia: The ARCH score. J. Thorac. Cardiovasc. Surg..

